# IMRT delivers lower radiation doses to dental structures than 3DRT in head and neck cancer patients

**DOI:** 10.1186/s13014-016-0694-7

**Published:** 2016-09-07

**Authors:** Eduardo Rodrigues Fregnani, Cláudia Joffily Parahyba, Karina Morais-Faria, Felipe Paiva Fonseca, Pedro Augusto Mendes Ramos, Fábio Yone de Moraes, Karina Gondim Moutinho da Conceição Vasconcelos, Gisela Menegussi, Alan Roger Santos-Silva, Thais B. Brandão

**Affiliations:** 1 Departments of Radiation Oncology and Oral Medicine, Sírio-Libanês Hospital, São Paulo, Brazil; 2 Department of Oral Diagnosis (Pathology and Semiology), Piracicaba Dental School, University of Campinas, Av. Limeira, 901 CEP 13414-903, Piracicaba, São Paulo Brazil; 3 Radiation Medicine Program, Princess Margaret Hospital, University of Toronto, Toronto, ON Canada; 4 Dental Oncology Service, Instituto do Câncer do Estado de São Paulo (ICESP), Faculdade de Medicinada Universidade de São Paulo, São Paulo, Brazil

**Keywords:** Intensity-modulated radiotherapy, 3D radiotherapy, Head and neck cancer, Teeth, Dental structures

## Abstract

**Background:**

Radiotherapy (RT) is frequently used in the treatment of head and neck cancer, but different side-effects are frequently reported, including a higher frequency of radiation-related caries, what may be consequence of direct radiation to dental tissue. The intensity-modulated radiotherapy (IMRT) was developed to improve tumor control and decrease patient’s morbidity by delivering radiation beams only to tumor shapes and sparing normal tissue. However, teeth are usually not included in IMRT plannings and the real efficacy of IMRT in the dental context has not been addressed. Therefore, the aim of this study is to assess whether IMRT delivers lower radiation doses to dental structures than conformal 3D radiotherapy (3DRT).

**Material and methods:**

Radiation dose delivery to dental structures of 80 patients treated for head and neck cancers (oral cavity, tongue, nasopharynx and oropharynx) with IMRT (40 patients) and 3DRT (40 patients) were assessed by individually contouring tooth crowns on patients’ treatment plans. Clinicopathological data were retrieved from patients’ medical files.

**Results:**

The average dose of radiation to teeth delivered by IMRT was significantly lower than with 3DRT (*p* = 0.007); however, only patients affected by nasopharynx and oral cavity cancers demonstrated significantly lower doses with IMRT (*p* = 0.012 and *p* = 0.011, respectively). Molars received more radiation with both 3DRT and IMRT, but the latter delivered significantly lower radiation in this group of teeth (*p* < 0.001), whereas no significant difference was found for the other dental groups. Maxillary teeth received lower doses than mandibular teeth, but only IMRT delivered significantly lower doses (*p* = 0.011 and *p* = 0.003). Ipsilateral teeth received higher doses than contralateral teeth with both techniques and IMRT delivered significantly lower radiation than 3DRT for contralateral dental structures (*p* < 0.001).

**Conclusion:**

IMRT delivered lower radiation doses to teeth than 3DRT, but only for some groups of patients and teeth, suggesting that this decrease was more likely due to the protection of other high risk organs, and was not enough to remove teeth from the zone of high risk for radiogenic disturbance (>30Gy).

## Introduction

Head and neck cancers (HNC) represent the sixth most common human malignancy in the world, with 442,760 new cases estimated for 2012 and a limited 5-years survival rate that achieves approximately 50 % in most of the series [1, 2]. Radiotherapy (RT) is frequently used in the treatment of HNC patients; however, treatment-associated side effects like mucositis, trismus, dysphagia, skin fibrosis, dysgeusia, osteonecrosis and xerostomia are found in patients mainly because of the lack of specificity of conventional radiation therapy that in addition to the tumor mass, also targets adjacent normal tissues [3, 4]. Although some authors claimed that radiation would not lead to direct dental damage [5], most of the studies demonstrate that RT can cause dental hard tissue disturbance, especially in its organic component, what may explain the higher frequency of radiation-related caries [6–11].

In an attempt to improve disease control and to decrease patients’ morbidity and toxicity, new RT planning techniques were developed. Intensity-modulated radiotherapy (IMRT) is a computerized optimization of the intensities of multiple radiation beams to strictly conform the treatment volume to tumor shapes, preserving adjacent normal structures, providing significantly better tumor target coverage and sparing sensitive normal tissue as compared with 3D radiotherapy (3DRT) in head and neck cancer [3, 12, 13]. Therefore, in this study we aimed to investigate if IMRT delivers lower radiation dose than 3DRT to dental structures.

## Material and methods

This study was approved by the Research Ethics Committee of the Institute of Teaching and Research of the Sírio-Libanês Hospital (Protocol No. 430.556) and of the Cancer Institute of São Paulo (Protocol No.171.972). In a 5-year period from 2010 to 2015 we retrospectively analyzed dental radiation dosage data of 80 HNC patients who underwent 3DRT (40 patients) and IMRT (40 patients) at the Cancer Institute of São Paulo and at the Sírio-Libanês Hospital, respectively.

Patients with clinicopathological information on age, gender, tumor location and clinical stage of the malignant disease and whose radiotherapy plans were available to be analyzed were included in this study. Patients were divided into four groups according to the primary tumor location (oral cavity, lateral border of tongue, oropharynx and nasopharynx). Patients on the 3DRT were treated in 6-MV linear accelerators on Synergy Platform (Elekta AB, Stockholm, Sweden) and received a mean radiation dose of 70Gy, whereas patients submitted to IMRT were treated on 6-18MV linear accelerator Novalis Tx Plataform on the Eclipse treatment planning system (Varian Medical Systems Inc., Palo Alto, CA, USA) and received a mean radiation dose of 66.7Gy.

Dosimetric analyses were performed for all patients by retrieving treatment planning and using calculation algorithms that incorporate tridimensional beam modeling on CMS XiO (Elekta CMS Software, St. Louis, MO) version 4.60 and Eclipse treatment planning system (Varian Medical Systems Inc., Palo Alto, CA, USA). Two previously trained dental oncologists, assisted by a medical physicist, reviewed each patient’s treatment plans based on axial slices of computed tomography scans to calculate the cumulative dose for the crowns of each group of radiated teeth, which were divided into incisors (anterior), premolars and molars. These groups were further classified into right and left sides to be evaluated according to their laterality in relation to the irradiated tumor location (ipsilateral and contralateral teeth). The mean dose delivered to each group of teeth was determined by individually contouring tooth crowns on the treatment planning systems and the average and maximum point of doses for each group were calculated.

A descriptive analysis was performed for the clinicopathological features and for maximum and average radiation doses received by dental structures. *T*-test was used to compare 3DRT and IMRT data. One-way ANOVA test was used for identifying significant differences in the radiation doses received by teeth according to dental groups and primary tumors treated with 3DRT or IMRT. When significance in this test was achieved, it was followed by Tukey’s Post-Hoc test to identify where significant differences were located. Minitab software version 17.3 and GraphPad Prism version 5.1 were used for statistical analyses and a *p*-value < 0.05 using a 95 % confidence interval was considered statistically significant.

## Results

Demographic features obtained from the 80 patients analyzed are described in Table 1. Of the 40 patients treated with 3DRT, 36 patients (90 %) also received concomitant chemotherapy, whereas 32 patients (80 %) of the 40 patients submitted to IMRT group also received chemotherapy. Radiotherapy was the primary treatment for 35 patients (87.5 %) and for 31 patients (77.5 %) of the 3DRT and IMRT groups, respectively, and adjuvant treatment for 5 patients (12.5 %) and for 9 patients (22.5 %) that received 3DRT and IMRT, respectively. In the 3DRT group 835 teeth were analyzed, whereas 1018 teeth were analyzed in the IMRT group.

**Table 1:** Clinicopathological features of the patients included in this study

Features	No. patients (%)	
	3DRT (*n* = 40)	IMRT (*n* = 40)
Sex		
Male	35 *(87.5 %)*	29 *(72.5 %)*
Female	5 *(12.5 %)*	11 *(27.5 %)*
Age (years)		
Mean	54.9	48.0
Range	25–81	14–78
Location		
Tongue	10 *(25.0 %)*	10 *(25.0 %)*
Oral Cavity	10 *(25.0 %)*	10 *(25.0 %)*
Oropharynx	10 *(25.0 %)*	10 *(25.0 %)*
Nasopharynx	10 *(25.0 %)*	10 *(25.0 %)*
T stage		
T1	6 *(15.0 %)*	9 *(22.5 %)*
T2	8 *(20.0 %)*	9 *(22.5 %)*
T3	9 *(22.5 %)*	14 *(35.0 %)*
T4	17 *(42.5 %)*	8 *(20.0 %)*
N stage		
N0	7 *(17.5 %)*	7 *(17.5 %)*
N1	10 *(25.0 %)*	9 *(22.5 %)*
N2	21 *(52.5 %)*	22 *(55.0 %)*
N3	2 *(5.0 %)*	2 *(5.0 %)*
M stage		
M0	40 *(100.0 %)*	40 *(100.0 %)*
M1	0 *(0.0 %)*	0 *(0.0 %)*

**Fig. 1:**
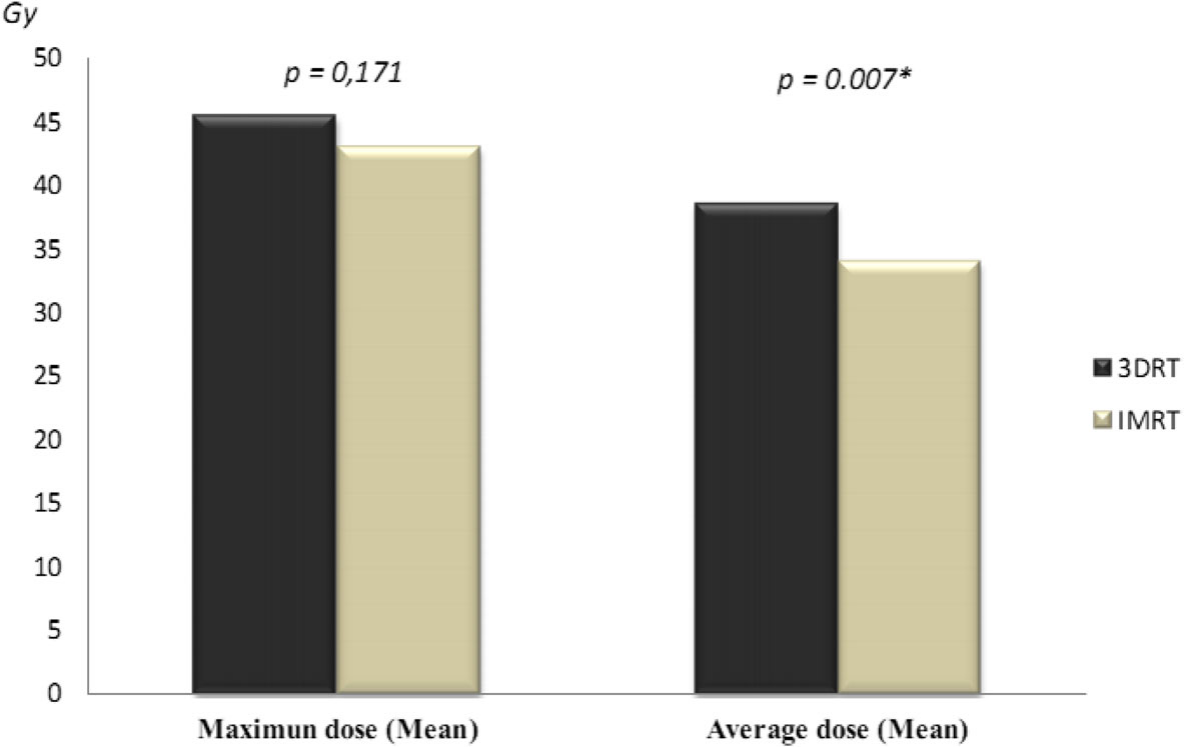
Mean of the maximum and of the average dental doses delivered by 3DRT and IMRT to patients treated for head and neck cancers. *Legend:* * Statistically significant difference according to *t*-test. 3DRT mean of the maximum dose 45.4 Gy (±24.8 Gy) and mean of the average dose 38.5 Gy (±24.5 Gy). IMRT mean of the maximum dose 43.0 Gy (±17.8 Gy) and mean of the average dose 34.0 Gy (±15.6 Gy)

**Fig. 2:**
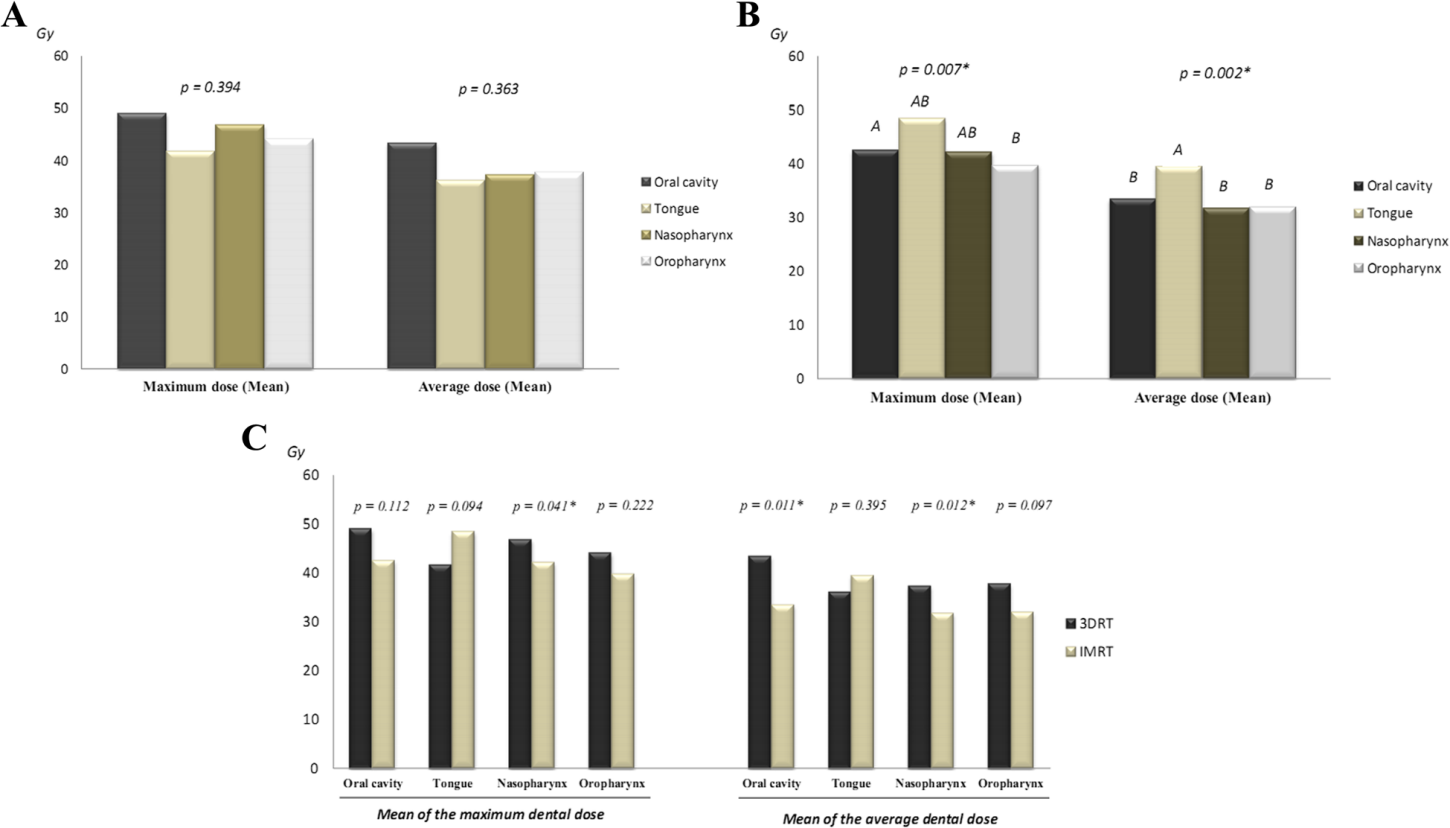
Mean of the maximum and of the average dental doses delivered by 3DRT and IMRT according to the location of the primary tumor treated. **a** There was no significant difference in the radiation doses (maximum and average) delivered by 3DRT to all HNC treated. *Legend:* There was no statistically significant difference according to One-way ANOVA test. Mean of the maximum doses: Oral cavity 49.02 Gy (±27.69 Gy), tongue 41.64 Gy (±25.34 Gy), nasopharynx 46.83 Gy (±16.51 Gy) and oropharynx 44.14 Gy (±28.06 Gy). Mean of the average doses: Oral cavity 43.39 Gy (±26.47 Gy), tongue 36.08 Gy (±25.51 Gy), nasopharynx 37.27 Gy (±17.14 Gy) and oropharynx 37.76 Gy (±27.69 Gy). **b** On the other hand, IMRT delivered significantly higher dental doses to patients treated for oral cavity if compared to oropharynx (maximum dose) cancer and to tongue if compared to all other cancers (average dose). *Legend:* * Statistically significant difference according to One-way ANOVA test. Different letters represent statistically different groups according to Tukey’s Post Hoc test. Mean of the maximum doses: Oral cavity 42.47 Gy (±18.07 Gy), tongue 48.36 Gy (±19.11 Gy), nasopharynx 42.06 Gy (±12.94 Gy) and oropharynx 39.67 Gy (±19.60 Gy). Mean of the average doses: Oral cavity 33.40 Gy (±16.37 Gy), tongue 39.41 Gy (±17.89 Gy), nasopharynx 31.68 Gy (±9.23 Gy) and oropharynx 31.95 Gy (±16.81 Gy). **c** Comparing both techniques we observed that 3DRT delivered significantly higher dental doses to patients treated for nasopharynx (maximum and average doses) and oral cavity (average dose) cancers than IMRT. *Legend:* * Statistically significant difference according to *t*-test

Evaluating the overall dental doses delivered by both techniques, we observed that the mean of the average doses received by patients submitted to 3DRT was significantly higher than that delivered by IMRT (*p* = 0.007), although there was no significant difference in the mean maximum doses delivered (*p* = 0.171) (Fig. 1). In addition, when primary tumor location was considered, there was no significant difference in dental doses received by patients affected by oral cavity, tongue, nasopharynx and oropharynx cancers treated with 3DRT (maximum dose *p* = 0.394 and average dose *p* = 0.363) (Fig. 2a). On the other hand, when treated with IMRT, patients affected by oral cavity cancer received significantly higher maximum doses than those affected by oropharynx cancer, whereas those affected by tongue cancer received significantly more radiation (average dose) than all other patients (variance analysis: maximum dose *p* = 0.007 and average dose *p* = 0.002) (Fig. 2b). Furthermore, we observed that patients affected by nasopharynx (maximum and average doses, *p* = 0.041 and *p* = 0.012, respectively) and oral cavity (average dose, *p* = 0.011) cancers treated with 3DRT received significantly more dental radiation than those treated with IMRT, whereas patients affected by tongue cancer treated with IMRT received more radiation than those treated with 3DRT, although this difference was not significant (maximum and average doses, *p* = 0.094 and *p* = 0.395) (Fig. 2c).

**Fig. 3:**
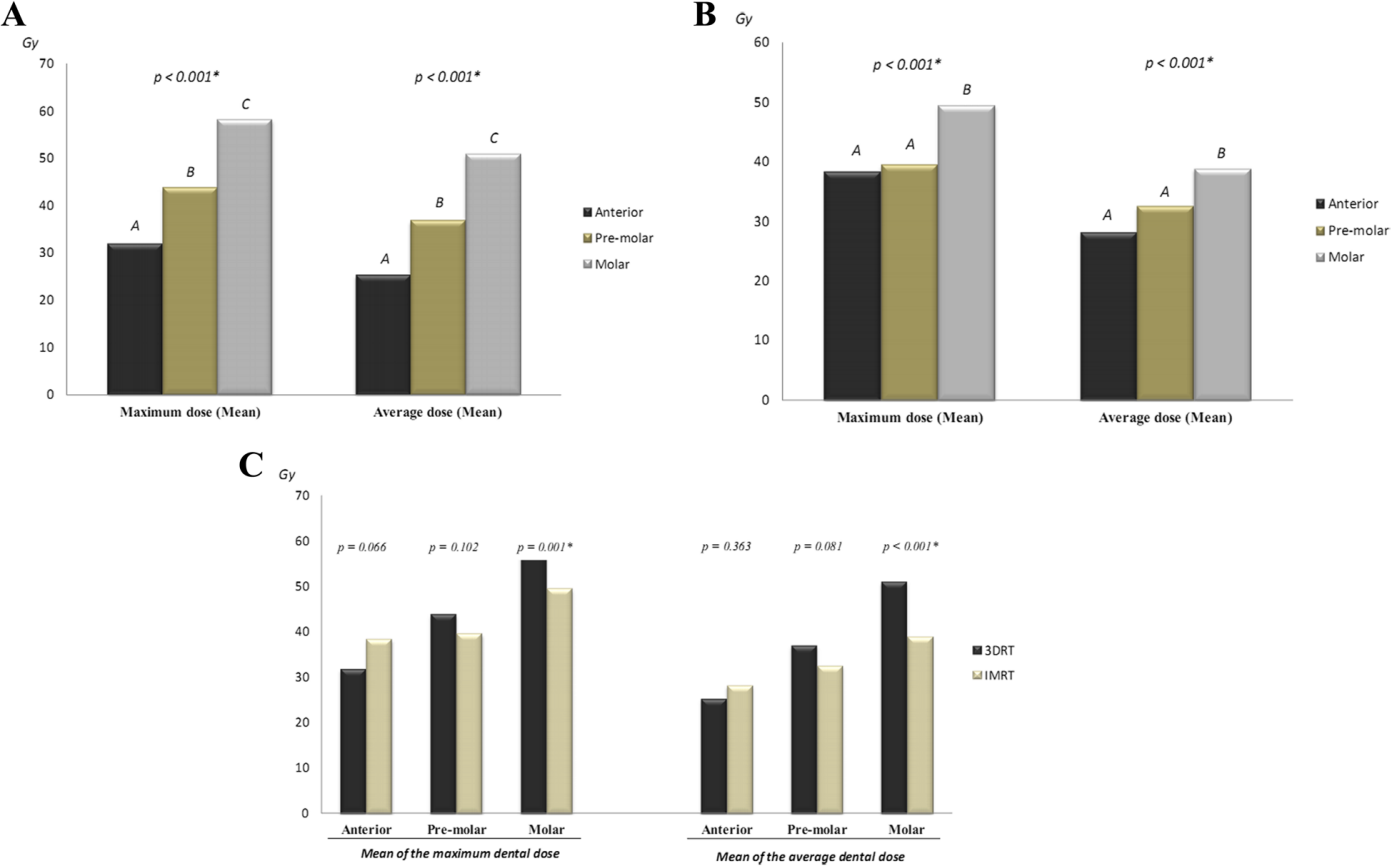
Mean of the maximum and of the average dental doses delivered by 3DRT and IMRT according to the dental groups investigated. **a** In the group of patients treated with 3DRT, molars received significantly higher doses (maximum and average doses) than pre-molar and anterior teeth, whereas pre-molars received significantly more radiation (maximum and average doses) than anterior teeth. *Legends:* * Statistically significant difference according to One-way ANOVA variance test. Different letters represent statistically different groups according to Tukey’s Post Hoc test. Mean of the maximum doses: Anteriors 31.74 Gy (±23.68 Gy), pre-molars 43.67 Gy (±23.49 Gy) and molars 58.02 Gy (±20.99 Gy). Mean of the average doses: Anteriors 25.20 Gy (±23.03 Gy), pre-molars 36.82 Gy (±23.24 Gy) and molars 50.75 Gy (±21.30 Gy). **b** In the group of patients treated with IMRT, molars also received significantly higher doses (maximum and average doses) than pre-molar and anterior teeth, but there was no significant difference between the last two groups. *Legends:* * Statistically significant difference according to One-way ANOVA variance test. Different letters represent statistically different groups according to Tukey’s Post Hoc test. Mean of the maximum doses: Anteriors 38.19 Gy (±17.30 Gy), pre-molars 39.39 Gy (±17.41 Gy) and molars 49.34 Gy (±16.63 Gy). Mean of the average doses: Anteriors 28.14 Gy (±14.35 Gy), pre-molars 32.43 Gy (±15.18 Gy) and molars 38.74 Gy (±15.36 Gy). **c** When we compared dental groups according to the technique used, we observed that in patients treated with 3DRT molars received significantly more radiation (maximum and average doses) than those treated with IMRT. *Legends:* * Statistically significant difference according to *t*-test

Radiation doses received by patients treated with 3DRT and IMRT according to dental groups and primary tumors are summarized in Table 2. 3DRT and IMRT both delivered higher radiations doses (maximum and average doses) to molars than to anterior teeth (variance analysis: *p* < 0.001 for 3DRT and IMRT) (Fig. 3a and b). When both techniques were compared, we found that molars of patients treated with IMRT received significantly less radiation than those of patients treated with 3DRT (*p* = 0.001 and *p* < 0.001, for maximum and average doses, respectively), whereas anterior teeth received more radiation with IMRT, but without statistical significance (*p* = 0.066 and *p* = 0.363, for maximum and average dose, respectively) (Fig. 3c).

**Table 2:** Mean of the maximum and mean of the average dosages (Gy) received by dental groups of patients submitted to IMRT and 3DRT protocols

	IMRT		3DRT	
	Mean maximum	Mean average	Mean maximum	Mean average
Tongue cancer				
Lower teeth				
Anterior	56.9	42.8	36.4	32.5
Pre-molars	51.5	43.6	56.9	49.5
Molars	50.8	42.3	64.7	59.1
Upper teeth				
Anterior	41.0	26.4	23.7	19.0
Pre-molars	42.7	36.1	20.9	16.8
Molars	47.5	36.7	43.7	37.7
Oral cavity cancer				
Lower teeth				
Anterior	47.0	34.6	48.9	43.7
Pre-molars	43.5	37.2	46.1	39.9
Molars	46.3	39.2	66.8	51.6
Upper teeth				
Anterior	38.4	24.0	37.2	34.3
Pre-molars	34.7	26.1	47.7	42.6
Molars	44.7	35.5	56.7	52.2
Oropharynx cancer				
Lower teeth				
Anterior	32.3	25.0	24.9	18.3
Pre-molars	36.5	31.3	40.6	34.5
Molars	50.5	41.0	57.0	52.1
Upper teeth				
Anterior	28.0	19.6	20.6	13.2
Pre-molars	34.1	28.4	47.5	39.9
Molars	46.8	36.6	55.7	49.2
Nasopharynx cancer				
Lower teeth				
Anterior	30.2	22.3	32.2	21.5
Pre-molars	37.1	28.0	44.9	37.5
Molars	54.6	39.9	65.6	57.2
Upper teeth				
Anterior	31.7	23.6	28.6	18.2
Pre-molars	35.1	28.7	40.7	30.3
Molars	52.5	38.8	60.8	50.3

Radiation doses delivered by 3DRT and IMRT to lower (mandibular) and upper (maxillary) teeth were also investigated. There was no significant difference in the doses delivered between both techniques to maxillary teeth (mean of the average *p* = 0.101 and mean of the maximum *p* = 0.521), but the mean average of radiation delivered by IMRT to mandibular teeth was significantly lower than 3DRT (mean of the average *p* = 0.043 and mean of the maximum *p* = 0.29) (Table 3). Maxillary teeth received lower doses of radiation than mandibular teeth, but significance was achieved only with IMRT technique (mean of the maximum *p* = 0.011 and mean of the average *p* = 0.003). By investigating maxillary and mandibular dosages according to primary tumors, we found statistically significant difference in the mean average and mean maximum doses delivered by 3DRT to tongue cancer (*p* = 0.0007 and *p* = 0.0003, respectively) and in the mean average delivered by IMRT to oral cavity cancers (*p* = 0.025) (Table 4).

**Table 3:** Radiation doses delivered to lower (maxillary) and upper (mandibular) teeth using 3DRT and IMRT

	3DRT		IMRT	
	Mean average	Mean maximum	Mean average	Mean maximum
Maxillary teeth	35.66 (±24.75)^aA^	42.74 (±24.87)^aA^	31.95 (±15.51)^aA^	41.21 (±18.01)^aA^
Mandibular teeth	41.34 (±24.07)^aA^	48.42 (±24.36)^aA^	36.79 (±15.65)^bB^	45.94 (±17.43)^bA^

Different lower case letters in columns mean statistically significant difference (*p* < 0.05) (*t*-test). In columns, compare 3DRT groups themselves and IMRT groups themselves

Different upper case letters in rows mean statistically significant difference (*p* < 0.05) (One way ANOVA test). In rows, compare mean of the averages themselves and mean of the maximums themselves

**Table 4:** Radiation doses delivered to lower (maxillary) and upper (mandibular) teeth using 3DRT and IMRT according to the primary site of the tumor

		Oral cavity		Tongue		Oropharynx		Nasopharynx	
		Mean average	Mean maximum	Mean average	Mean maximum	Mean average	Mean maximum	Mean average	Mean maximum
3DRT	Maxilla	43.38 (±27.27)^aA^	48,17 (±27.24)^aA^	25,31 (±24.36)^aB^	30,26 (±24.81)^aB^	37,75 (±27.36)^aAB^	44,85 (±27.28)^aA^	34,83 (±17.21)^aAB^	45,28 (±17.02)^aAB^
	Mandible	43.41 (±26.01)^aA^	50,86 (±28.29)^aA^	47,64 (±21.66)^bA^	53,51 (±19.59)^bA^	36,99 (±28.47)^aA^	43,47 (±29.08)^aA^	40,13 (±16.85)^aA^	48,66 (±15.95)^aA^
IMRT	Maxilla	29.58 (±15.02)^aA^	39.76 (±17.58)^aA^	35.63 (±19.88)^aA^	44.07 (±21.33)^aA^	29.93 (±16.06)^aA^	38.04 (±19.77)^aA^	31.75 (±8.99)^aA^	41.38 (±12.24)^aA^
	Mandible	37.31 (±16.93)^bAB^	45.23 (±18.35)^aAB^	42.93 (±15.20)^aAB^	52.37 (±15.99)^aAB^	33.92 (±17.44)^aA^	41.28 (±19.51)^aA^	31.62 (±9.56)^aA^	42.74 (±13.69)^aA^

Different lower case letters in columns mean statistically significant difference (*p* < 0.05) (*t*-test). In columns, compare 3DRT groups themselves and IMRT groups themselves

Different upper case letters in rows mean statistically significant difference (*p* < 0.05) (One way ANOVA test + Tukey’s post-hoc test). In rows, compare mean of the averages themselves and mean of the maximums themselves

**Fig. 4:**
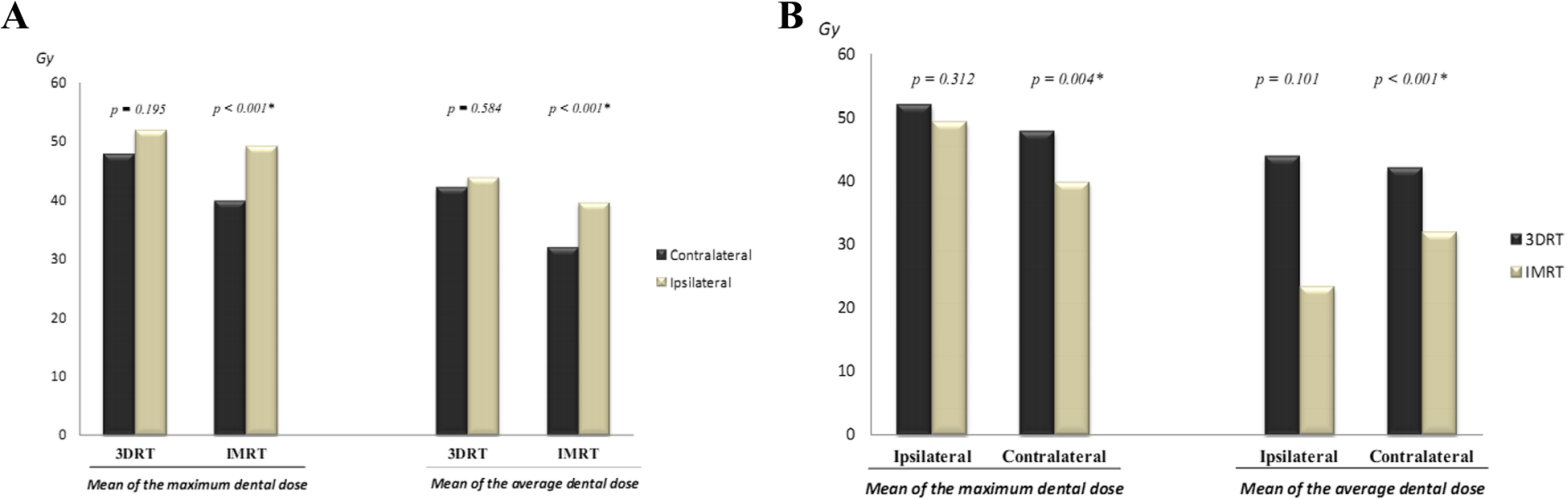
Mean of the maximum and of the average dental doses delivered by 3DRT and IMRT according to the dental laterality in regard to primary tumors. **a** Teeth located ipsilateral to primary tumor received higher doses of radiation than their contralateral counterparts; however, this difference was significantly different only for those patients treated with IMRT. *Legends:* * Statistically significant difference according to *t*-test. Mean of the maximum doses: Contralateral teeth (3DRT) 47.70 Gy (±23.30 Gy) and ipsilateral teeth (3DRT) 51.90 Gy (±23.60 Gy); contralateral teeth (IMRT) 39.70 Gy (±17.30 Gy) and ipsilateral teeth (IMRT) 49.20 Gy (± 17.00 Gy). Mean of the average doses: Contralateral teeth (3DRT) 42.00 Gy (±23.60 Gy) and ipsilateral teeth (3DRT) 43.80 Gy (±23.30 Gy); contralateral teeth (IMRT) 31.80 Gy (±14.70 Gy) and ipsilateral teeth (IMRT) 39.50 Gy (± 15.50 Gy). **b** When we compared both techniques we observed that contralateral teeth of patients treated with 3DRT received statistically more radiation than those treated with IMRT. *Legends:* * Statistically significant difference according to *t*-test

Ipsilateral dental groups received higher doses than contralateral teeth submitted to both 3DRT and IMRT, although statistical significance was achieved only for patients treated with latter (*p* < 0.001 for both maximum and average doses) (Fig. 4a). Comparing both techniques, IMRT delivered less radiation to both ipsilateral and contralateral teeth than 3DRT; however, statistical significance was obtained only for contralateral teeth (*p* = 0.004 and *p* < 0.001, for maximum and average doses, respectively) (Fig. 4b).

## Discussion

HNC is an aggressive human disease with unsatisfactory 5-years survival rates. Improvements in the radiotherapy delivery techniques to increase tumor control and decrease patients’ toxicity and morbidity led to the development of IMRT that constrains radiation beams to tumor volume and limits the involvement of adjacent normal tissues, tightly modulating the radiation intensity for each area of the target [12]. Consequently, patients submitted to IMRT have lower risks of osteoradionecrosis of the jaws, less xerostomia, less pain and lower incidence of mucositis when compared to those receiving 3DRT, with an improved global quality of life [14, 15], what affirms the superiority of IMRT to obtain less RT related toxicity [16].

Radiation-related caries are a common complication of anti-neoplastic therapy that mainly affect the vestibular, cervical and oclusal/incisional dental surfaces and cause extensive enamel loss that gives rise to an extensive brownish discoloration of the teeth. Our group has previously demonstrated that pulpal components of irradiated teeth are not directly affected by radiation [5, 17], supporting the hypothesis that radiation-related caries would primarily represent a consequence of modifications in saliva production by irradiated salivary glands that would alter saliva composition, reduce oral clearance and cause significant changes of the oral microflora, increasing the concentration of acidogenic and cariogenic microorganisms [18]. However, different studies have documented direct alterations in the enamel, dentin and enamel-dentin junction (EDJ), what would be responsible for or facilitate the development of radiation-related caries [6, 8]. Therefore, according to these studies, protecting dental hard tissues from direct radiation would represent an important objective during head and neck radiotherapy.

Some studies attempted to demonstrate the radiation dosage received by dental structures and tooth-bearing regions of patients that received 3DRT and IMRT for HNC treatment [4, 19, 20]; however, there seems to be no previous analysis comparing the dental radiation doses delivered by both approaches. In this context, we demonstrated in this survey that patients treated with IMRT received significantly lower doses of dental radiation than those treated with 3DRT. However, when we considered the location of primary tumors only patients affected by nasopharynx and oral cavity cancers demonstrated statistically significant lower doses when treated with IMRT. In our opinion, this finding more likely represents an indirect dental benefit, because teeth are not included in the IMRT constrained areas during radiation planning, what allow us to further speculate that the lower dental radiation doses obtained with IMRT only for these patients would be an indirect advantage of the protection of other high risk organs like the salivary glands. Moreover, the contradictory result found for tongue cancer that revealed lower dental doses with 3DRT also corroborate our hypothesis of indirect benefit of IMRT to dental structures, since we believe that patients affected by any HNC should have demonstrated a homogeneous lower dental dose with IMRT if teeth were spared in the radiation planning.

In our study, with no dental constrain during IMRT planning, we observed that only 10 out of 23 dental groups analyzed received less than 30Gy as an average radiation dose, and only one dental group (upper anterior teeth of oropharynx cancer patients) received less than 30Gy as the mean maximum dose; moreover, the total amount of dental radiation delivered by IMRT was of 34Gy and 43Gy (mean of the average and mean of the maximum doses). These results show that even using IMRT, dental structures were exposed to radiation doses above the cut off value of 30Gy proposed by Walker et al. [6] that would expose patients to a 2–3 times higher risk for direct dental hard tissue disturbance.

This observation demonstrates that by not including teeth in the constrained plans of IMRT, the significant decrease in the radiation doses obtained when compared to 3DRT were not enough to remove teeth from the zone of high risk for radiogenic disruption. This rationale explains the findings described by Duarte et al. [21] who failed to obtain a significant difference in the number of caries developed by patients treated by 3DRT and IMRT, by Kataoka et al. [17] and Kataoka et al. [22] who also failed to demonstrate any significant difference on dental pulp sensitivity of patients treated with IMRT and 3DRT and by Beesley et al. [23] that found no difference in tooth loss between both modalities. In addition, Gomez et al. [24] also observed that 17 % of their sample (168 patients) experienced a dental event (caries or tooth loss) during follow-up after IMRT therapy.

All these results suggest that teeth must be included in the IMRT planning to decrease dental radiation exposure and to obtain all benefits of this approach in the dental context. This assumption is also supported by Verdonck et al. [25] who reported lower radiation doses in the anterior mandible of patients affected by oropharynx cancer when IMRT planning was appropriately performed, allowing the use of anterior dental implants in these patients.

In this study we also found significantly higher doses of radiation in posterior teeth using both 3DRT and IMRT. This finding is in accordance to Bak et al. [26] that using IMRT obtained higher doses of radiation in molars of patients affected by cancers from the base of the tongue, tonsil and hypopharynx. Similarly, Hansen et al. [27] and Parahyba et al. [20] also obtained higher radiation values for molars, reporting that tumor size is very important to predict the amount of radiation delivered to tooth-bearing regions, since large tumors revealed high doses in the entire mandible, for this reason we attempted to gather tumors with as much similar TNM stage as possible.

On the other hand, we observed that only molars demonstrated significantly lower dental doses with IMRT than with 3DRT, whereas anterior teeth presented higher, although non-significant, radiation doses with IMRT. Once again, this variability in the doses received by different dental groups also supports our hypothesis of an indirect benefit obtained with IMRT.

Mandibular teeth seems to be more irradiated than maxillary teeth irrespective of primary tumor site when using 3DRT [4], what could also be demonstrated in our study. However, a recent report demonstrated lower mandibular doses in patients affected by nasopharynx cancer treated by IMRT [20], suggesting that using this technique primary tumor location would determine the most irradiated jaw. This finding was also observed in our sample, since only patients affected by nasopharynx cancer exhibited a slightly lower mean average radiation in mandibular teeth if compared to maxillary ones. IMRT also provided less radiation doses to teeth located either ipsilateral or contralateral in relation to primary tumor, but with a significant difference to 3DRT only for those teeth positioned contralateral to the lesion. The lower radiation doses observed in contralateral teeth had also been previously described [4, 20].

## Conclusion

In conclusion, we showed in this study that even when teeth are not constrained for radiation exposal during IMRT treatment for HNC, it provides significantly lower radiation doses to dental hard tissues if compared to 3DRT, but only for some groups of patients and teeth, what may represent an indirect advantage as a consequence of the protection of other high risk organs. However, these lower radiation doses were not enough to remove teeth from the band of high risk for radiogenic dental disruptions (> 30Gy), suggesting that teeth must be included in the IMRT sparring plans so that we can benefit from the advantages of this technique for dental health maintenance. Nevertheless, studies comparing the radiation doses delivered to teeth with and without including dental structures in IMRT constrained plans remain to be performed to confirm this hypothesis.
